# Patellofemoral pain in general practice: the incidence and management

**DOI:** 10.1093/fampra/cmad087

**Published:** 2023-09-05

**Authors:** Guido J van Leeuwen, Evelien I T de Schepper, Patrick J E Bindels, Sita M A Bierma-Zeinstra, Marienke van Middelkoop

**Affiliations:** Department of General Practice, Erasmus MC University Medical Center, Rotterdam, The Netherlands; Department of General Practice, Erasmus MC University Medical Center, Rotterdam, The Netherlands; Department of General Practice, Erasmus MC University Medical Center, Rotterdam, The Netherlands; Department of General Practice, Erasmus MC University Medical Center, Rotterdam, The Netherlands; Department of Orthopedics and Sports Medicine, Erasmus MC University Medical Center, Rotterdam, The Netherlands; Department of General Practice, Erasmus MC University Medical Center, Rotterdam, The Netherlands

**Keywords:** adolescents, cohort studies, epidemiology, general practice, incidence, knee, management, patellofemoral pain, primary health care

## Abstract

**Background:**

Patellofemoral pain (PFP) is a nontraumatic knee problem primarily observed in physically active adolescents. The objective of this study was to determine the incidence and management of PFP in children and adolescents in general practice

**Methods:**

A retrospective cohort study was conducted using a regional primary care database containing full electronic health records of over 300,000 patients. Patients with a new PFP diagnosis between the years 2013 and 2019 were extracted using a search algorithm based on International Classification of Primary Health Care coding and search terms in free text. Data on the management of PFP were manually checked and analysed. In addition, a sub-analysis for chronic and nonchronic PFP patients was performed.

**Results:**

The mean incidence of PFP over the study period was 3.4 (95% CI 3.2–3.6) per 1,000 person years in the age group of 7–24 years. Girls had a higher incidence rate (4.6 [95% CI 4.3–5.0]) compared to boys (2.3 [95% CI 2.1–2.5]). Peak incidence was at age 13 years for both sexes. The most commonly applied management strategy was advice (55.1%), followed by referral to physiotherapy (28.2%), analgesics prescription (10.4%), and referral to the orthopaedic surgeon (8.9%). No differences were found in age, sex, and treatment between chronic and nonchronic PFP patients.

**Conclusions:**

The average Dutch general practitioner sees approximately 1.4 new child or adolescent with PFP per year. Overall management strategies were in concordance with current Dutch general practice guideline on nontraumatic knee problems. More insight should be gained in the population with chronic complaints.

**Key messages**

- The average Dutch general practitioner (GP) sees approximately 1.4 new young patellofemoral pain (PFP) patients per year.
- The incidence of PFP in Dutch general practice is 3.4 per 1,000 person years.
- PFP has a higher incidence in girls than boys.
- Most patients visit the GP only once.
- One in 3 patients is referred to the physiotherapist.
- No difference between chronic and nonchronic PFP patients in age, sex, or treatment.

## Background

In children and adolescents, musculoskeletal (MSK) problems can have a significant impact on the normal daily routine of school, social, and sports participation.^1–3^ Currently there still is a limited understanding of the epidemiology, burden, and treatment of MSK problems in children and adolescents.^4^ Even though chronic or recurrent pain occurs in up to 25% of the children and adolescents.^5–7^

In adolescents, knee problems (32%) are one of the most frequently reported MSK problems.^8^ One of the most common forms of knee pain is patellofemoral pain (PFP).^9^ In research the reported incidence and prevalence of PFP varies greatly, possibly due to the evaluation of different populations and the inconsistency and lack of high-quality evidence on which assessment and diagnosis of PFP are based.^10,11^ Albeit, a recent systematic review^12^ reports a PFP incidence of 9.7 per 1,000 person years and an annual prevalence in the general population of 22.7%, with an even higher prevalence in adolescents (28.9%). However, the studies included in this review consisted of all age categories and were predominantly done in specific highly active populations such as military recruits and (elite) athletes. PFP is thought to have the highest incidence and prevalence in active adolescents,^13,14^ elite athletes^15^ and occurs more often in girls than boys.^16,17^ PFP can be characterized as a nontraumatic knee problem with diffuse anterior retro-patellar pain and crepitation mainly during activities that load the joint such as running, jumping, squatting, climbing, and descending stairs.^9^ Palpation of the patella, the patellofemoral compression test, and resisted knee extension are often painful during physical examination.^16,18,19^ Physical overuse, trauma, and anatomic factors appear to be predisposing components in the development of PFP.^20,21^ It is generally thought that these type of knee problems are self-limiting in adolescents and cease to exist after puberty and skeletal maturation.^22,23^ Nevertheless, studies show that adolescents with knee pain have a high risk of persistent knee pain, with an even higher risk for adolescents with PFP.^7,24,25^ This is reflected by 1 study that reported persistent pain in almost 40% of the adolescents with PFP after 6 years.^25^ It has even been suggested that adolescent PFP may lead to patellofemoral osteoarthritis,^20,26,27^ but currently there is no clear and definitive evidence for this suggested disease trajectory.

According to the current Dutch general practice guideline (Nederlands Huisartsen Genootschap [NHG]) on nontraumatic knee problems, the diagnosis of PFP is purely based on anamnesis and physical examination.^28^ Treatment for PFP initially consisted of advice, patient education, rest, and in some cases short-term use of analgesics.^12,21,28^ In 2013, as a result of various studies, physical therapy, and strengthening exercises were included in the NHG guideline as a recommendation for the treatment of PFP.^29,30^

The objective of this study was to determine the incidence and prevalence, including the distribution of age and sex of PFP amongst children and adolescents in Dutch general practice. Furthermore, this study aims to gain insight into the different types of treatment strategies applied by GPs, the duration of complaints and to compare management between chronic and nonchronic patients.

## Methods

### Design and setting

A retrospective cohort study was conducted in the Rijnmond Primary Care Database (RPCD).^31^ The database contains anonymous longitudinal data on demographics, symptoms, and diagnosis, correspondence to and from secondary care and drug prescriptions of over 200,000 participants in the greater area of the city of Rotterdam. The RPCD is a regional derivative from the nationwide Integrated Primary Care Information database.^32^ In the Netherlands it is obligated for citizens to be registered with a general practitioner (GP) and in case complaints require medical care the GP is contacted first, also for referrals to secondary care.

### Study cohort

The study population consisted of patients aged 7–24 years with a new diagnosis of PFP, seen by the GP in the RPCD between 1 January 2013 and 31 December 2019. During the study, 46 general practices were included in the database including 43,573 patients aged between 7 and 24 years. Patients with at least 12 months of valid database time were eligible to be included in the cohort, provided that they were never diagnosed with PFP in their database history. Diagnoses of PFP were identified using the International Classification for Primary Care (ICPC) coding and with supporting keywords in the free text.^33^ Patients were considered to have PFP if they received the ICPC code L99.07 (Retropatellar chondropathy/Patellofemoral syndrome) or L15 (Knee symptoms/complaint)in combination with the words or abbreviations ‘Patell’, ‘PFP’, ‘Chondropathy’ in the free text. The final algorithm excluded hits that were combined with terms of negation (ex. not or no) and hits where the GP combined the ICPC codes with a specific diagnosis other than PFP. Possible unclear cases were inspected by a senior researcher (ES), and final decisions whether or not to include the case as PFP were based on consensus.

### Data extraction

The full medical files were examined from the consultation date of the initial diagnosis until the last consultation of the episode. Cases were considered to be a true PFP case if the GP defined the consultation as PFP or considered it as 1 of several possible diagnoses (i.e. in the differential diagnosis). Cases where the PFP diagnosis did not match our definition were excluded from further analyses. For each patient, information on date of birth and sex were extracted. Furthermore, the interventions applied by the GP, the referrals, and the number of consultations with the GP for 1 episode of complaints related to PFP were extracted from the full medical record by 1 of the authors (GvL). After a preliminary check of the dataset, the following treatment strategies were registered in the final analysis of the cases: advice (e.g. patient education, rest and wait and see), medication (analgesics), and referral comprising of imaging (X-ray, ultrasonography, or Magnetic resonance imaging [MRI]), physiotherapist, orthopaedics, sports medicine, and other (para)medical specialists (i.e. podiatry). Multiple treatments could be administered per consultation. Furthermore, it was also registered if the GP mentioned muscle strengthening exercises in the patients who received advice and the amount of NSAID prescriptions in the patients who received medication. For each patient, the date of the first consultation and each consequent consultations were extracted. Telephone consultations were also counted as consultations, provided that the GP had contact with the patient and discussed the diagnosis or treatment of PFP. Having chronic PFP complaints,^34^ was defined as having multiple consultations for PFP with minimally more than 3 months between the first and any of the following consultations.

### Statistics

The incidence rate of PFP per age group 7–24 years was determined by dividing the number of PFP cases by the total number of person years of follow-up in all patients aged 7–24 years included in RPCD and expressed per 1.000 person years. Ninety-five percent confidence intervals (95% CI) were calculated using Poisson distribution. The incidence and the number of patients in an average Dutch GP practice (2,000 patients of which 406 patients aged 7–24 years) were used to calculate the number of new PFP patients per year. Descriptive statistics were used to describe the types of treatment strategies applied and the number of consultations of patients with PFP. The same analyses were also done for the chronic and nonchronic PFP group separately. All analyses were performed using SPSS version 25, R Studio was used for Poisson distribution.

## Results

### General characteristics

**Fig. 1.**
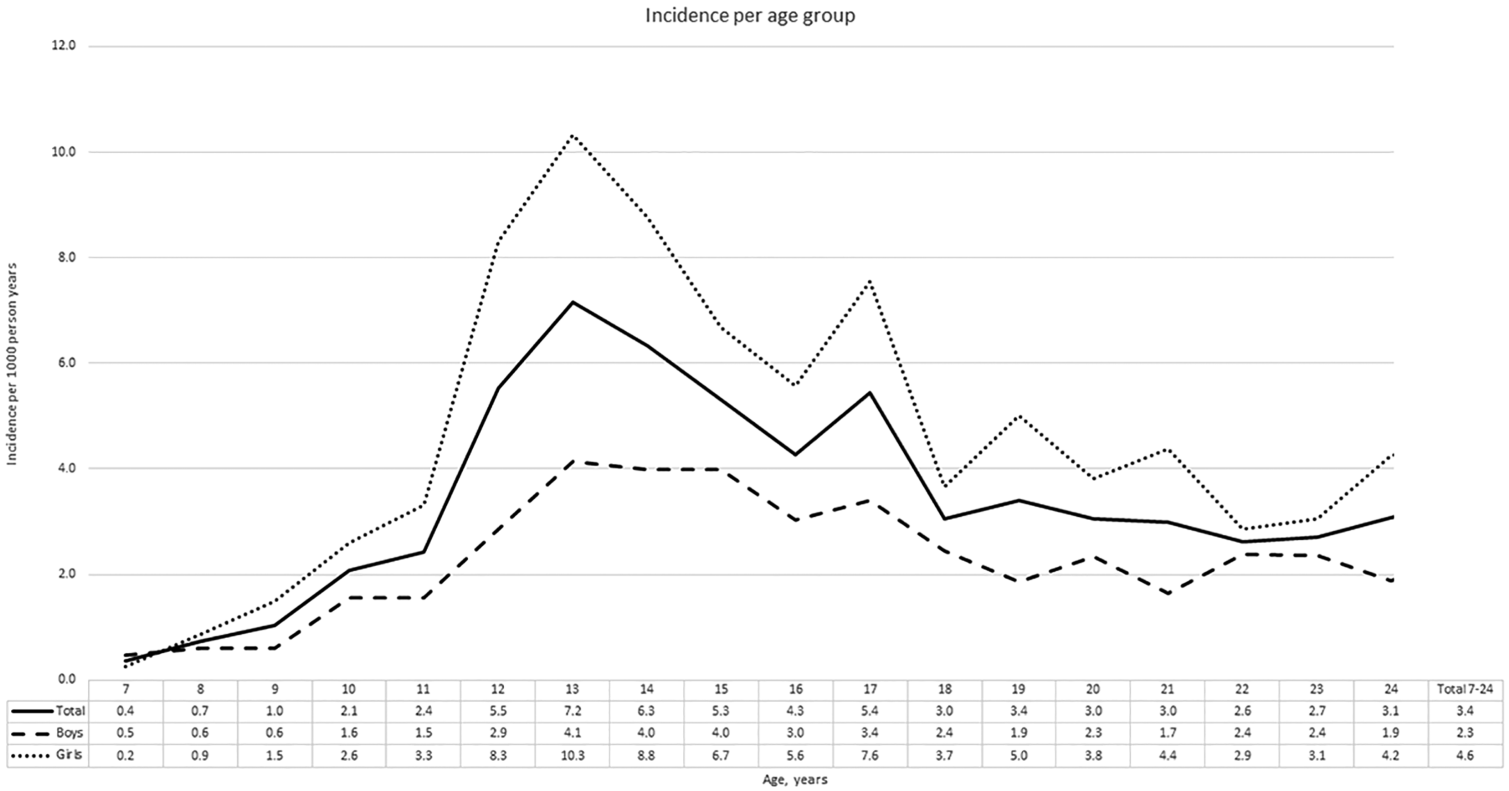
Incidence of patellofemoral pain per age group during the years 2013–2019. The incidence of patellofemoral pain per age group (7–24 years).

In the years 2013–2019, the search strategy in the database revealed 1,359 patients aged 7–24 years at the moment of diagnosis. After manual validation, 1,030 true PFP patients remained with a median follow-up time of 1043 (P_25–75_ 507–1685) days. In this population, overall incidence was 3.4 (95% CI 3.2–3.6) per 1,000 person years. Almost two-thirds (66.1%) of the PFP patients were girls with an incidence of 4.6 (95% CI 4.3–5.0) per 1,000 person years compared to 2.3 (95% CI 2.1–2.5) in boys. Within the period 2013–2019, the yearly incidence was stable with overlapping 95% CI’s. The incident PFP population had a median age of 15 years (P_25–75_ 13–19), the median age for girls was 15 (P_25–75_ 13–19) and for boys 16 (P_25–75_ 13–20). Peak incidences for girls and boys were respectively 10.3 (95% CI 8.2–12.8) and 4.1 (95% CI 2.9–5.7) per 1,000 person years at age 13 years (Fig. 1).

### Consultations and management strategies

**Fig. 2.**
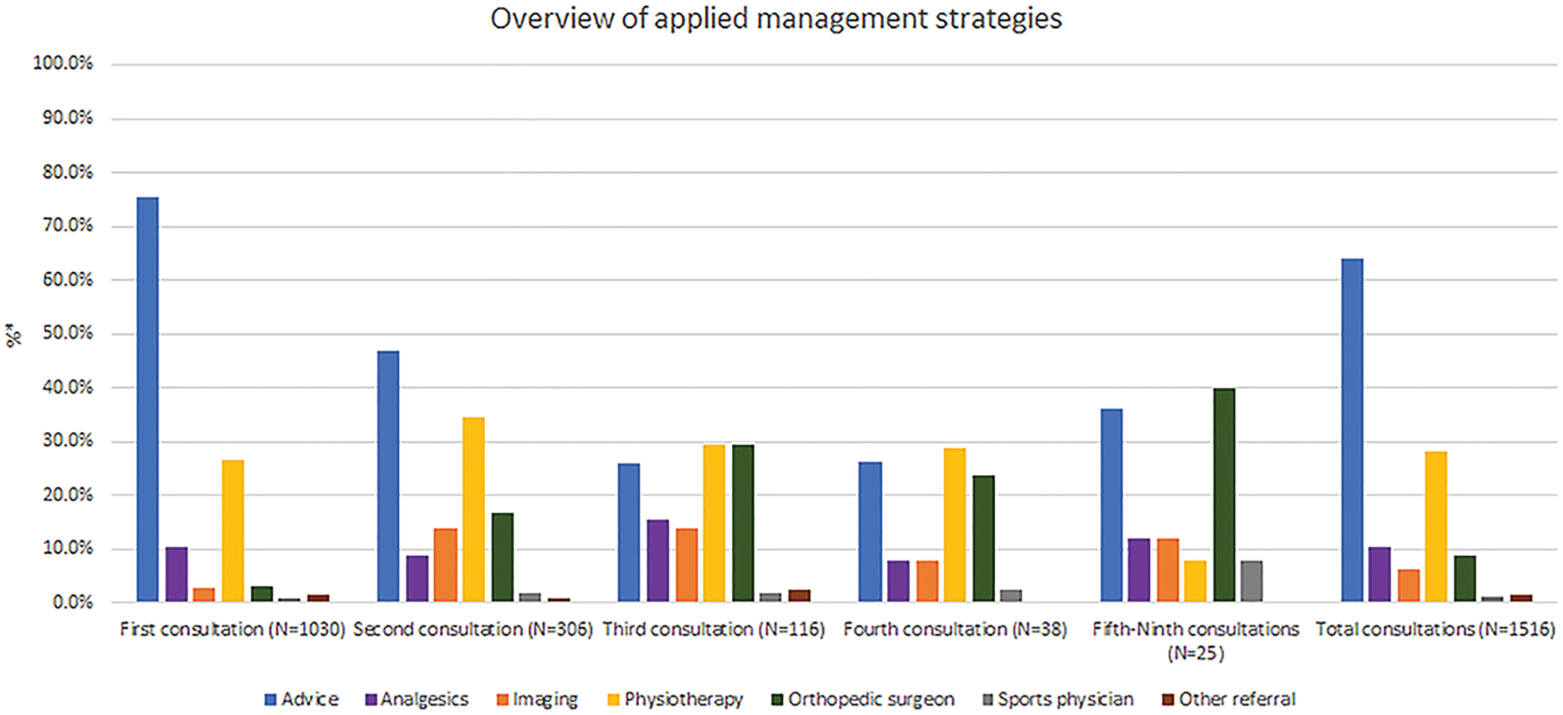
Overview of applied management strategies during all consultations. Overview of the different applied management strategies in all consultations and in the total amount of consultations (range 1–9). Because of a low number of patients consultation 5–9 were combined. A detailed overview with exact percentages can be found in Supplementary Table 1. *Patients could receive more than one intervention per consultation, therefore the total of patients in these cells does not add up to 100%.).

The 1,030 incident cases resulted in a total of 1,516 consultations (range 1–9). The PFP episode was limited to 1 consultation in 70.3% (*n* = 724) and limited to 4 consultations in 98.3% (*n* = 1,013) of the patients. In Fig. 2, we present an overview of the applied management strategies during all consultations. Advice was the most frequently applied management strategy by the GP (75.5%) during the first consultation of all episodes, followed by physiotherapy (26.6%) and analgesics (17.5%). Referrals were to the orthopaedic surgeon (3.0%), imaging (2.8%), other (para)medical specialists (1.6%), and sports physician (0.8%). Additionally, the GP explicitly mentioned the added benefit of muscle-strengthening exercises in 28.7% of patients who received advice and in patients who received analgesics, 55% received NSAIDS during the first consultation. Supplementary Table 1a provides a more detailed of overview applied management strategies during all consultations.

### Follow-up consultations

**Fig. 3.**
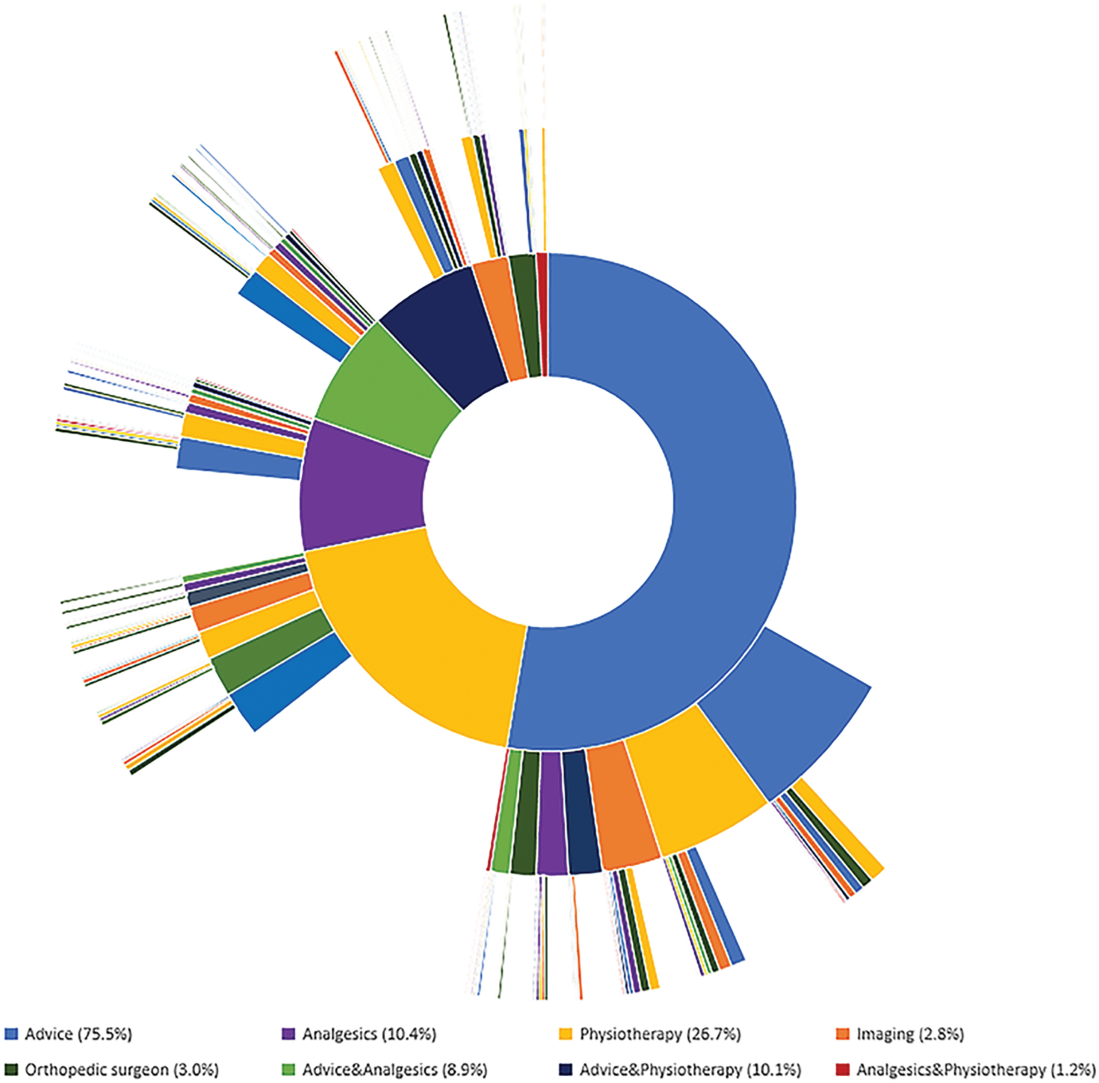
Hierarchical visualization of the management of patellofemoral pain per consultation (2013–2019). The inner circle represents the policy in the first consultation (*N* = 1,030 patients). The second circle represents the policy in the second consultation (*N* = 306) and the third circle represents the policy in the third consultation (*N* = 116). Percentages are based on the management in the first consultation. Patients could be treated according to more than one policy; consequently, the total percentage may exceed 100%.

In total, 306 (29.7%) patients had multiple consultations (range 2–9) for PFP with their GP after initial diagnosis: 72.5% were girls with a median age of 15 years (P_25–75_ 13–19). In these patients, the median time between the first and second consultation was 323 (P_25–75_ 105–657) days. Figure 2 and Supplementary Table 1a show that advice is the most often applied management strategy until the third consultation when referral to the physiotherapist and orthopaedic surgeon were most often applied. Generally, the rate of referral for imaging or a medical specialist, except referral to the physiotherapist, rises with each following consultation. For example, the rate of referral for imaging rose from 2.8% to around 14% at the second or third consultation, while referral to the physiotherapist was stable with approximately 30% at each consultation. Figure 3 provides an overview on the flow of patients from first to third consultation. Patients who were referred for imaging or received medication had the highest percentage of second consultations, respectively 55.2% and 43.0%. Patients that received a referral to the orthopaedic surgeon had the lowest percentage of second consultations (19.4%).

In total, 234 (22.7%) of the patients had chronic complaints (≥3 months) of which 73.1% (*n* = 171) were girls compared to 64.1% in the nonchronic population (*n* = 796). Chronic patients had a median age of 15 (P_25–75_ 13–18) compared to 16 (P_25–75_ 13–20) years in the nonchronic patients. Supplementary Table 1b and c gives an overview of the different applied management strategies in both patient groups. No differences were seen in the rates of applied management strategies between both patient groups and also the main results.

## Discussion

This study is the first retrospective cohort study based on longitudinal population data that provides insight in the incidence of PFP in general practice as well as the different management strategies applied in this population. In the age group 7–24 years, we found an incidence of 3.4 per 1,000 person years, with a higher incidence for girls than boys. This incidence indicates that an average GP practice in the Netherlands sees about 1.4 new young PFP patients per year. The GP was visited only once by most patients (70.3%). At first consultation most patients received advice, including rest and a wait-and-see policy, followed by a referral to the physiotherapist as a management strategy. Only few patients were referred to the orthopaedic surgeon (3.0%) or imaging (2.8%) during the first consultation.

### Strengths and limitations

This is the first study to examine the incidence of PFP based on medical files of a large group of patients in general practice. It provides a representative overview on the incidence and management in general practice.

GP medical records have certain limitations since they are not primarily intended for data collection, for example possible selection bias as a results of the diagnostic accuracy of the GP and dependence on reporting in the medical file by the GP.^35^ Multiple ICPC codes and free text terms to identify PFP cases were used to limit the possible underestimation of the overall incidence due to limited medical notes or nonuniform ICPC coding.^36^ Furthermore, it was not possible to take into account the factors that influence the decision making of the GP, gain insight into the process of shared decision making or draw conclusions about the effectiveness of the administered treatment. Moreover, the duration of complaints before the first consultation at the GP for each patient was not available to us. Thus, we defined chronic complaints as patients with at least 3 months^34^ between the first and 1 of their subsequent consultations. This however could have led to an underestimation of the amount of chronic patients since in theory patients with only 1 consultation could already have had complaints for more than 3 months. Unfortunately in this study, it is not possible to estimate the amount of patients in whom this may have occurred. In previous research, the duration of complaint before presentation varies greatly, ranging from most patients presenting within 3 months^24,25,29^ to over a year^2,7,17^ after the start of complaints. Lastly, since paracetamol and low-dosage NSAIDs are over-the-counter analgesics in the Netherlands, the amount of prescriptions in this study may not fully reflect the total analgesics used for this complaint in the study population.

### Comparison with existing literature

We found an incidence of 3.4 per 1,000 person years for PFP, which is lower than a previously reported incidence of 9.7 per 1,000 person years.^12^ This higher incidence is a result of the included studies in the systematic review, which were performed in all age categories and predominantly in specific highly active populations such as military recruits and (elite) athletes. Our findings showed a higher incidence of PFP and a lower median age in girls compared to boys. This is in concordance with previous literature where PFP is more often seen in girls and complaints present at an earlier age than boys.^16,17^ MSK complaints and specifically nontraumatic knee complaints are likely to present earlier in girls because of the earlier onset of puberty and skeletal maturation.^37^

In concordance with the current NHG guideline for nontraumatic knee complaints^28^ the most often administered management strategies were advice and referral to physiotherapy. In this study, the rate of referral to physiotherapy rose from 23.6% in 2013 to 33.6% in 2014. Possibly coinciding with the incorporation of physiotherapy as a treatment option for PFP in the NHG guideline^28^ in 2013. After 2014 the rate of referral remained relatively stable around thirty percent in the remaining study period. The rate of referral in our study could be an underestimation of the actual rate of patients that visit the physiotherapist for PFP since the physiotherapist is directly accessible for patients in the Netherlands.

Furthermore, the GP explicitly mentioned the added benefit of muscle-strengthening exercises in approximately 30% of patients who were managed with advice. Thus, in our study, potentially more than half of patients are managed with physical therapy and muscle-strengthening exercises.

Nevertheless, the actual content of management by the physiotherapist was not available, so it is unclear if they were managed with specific PFP exercise therapy.^29,30^ Additionally, research has shown that muscle-strengthening exercises without supervision of a physiotherapist leads to lower adherence resulting in a lower efficacy of treatment and odds of recovery.^38^ The rate of referral to the orthopaedic surgeon remained fairly stable with around 7% per year during the study period, despite the availability of physiotherapy as a new management strategy. More awareness by the GP, by better implementation of available knowledge and the Dutch guidelines, may possibly further increase the referral rate to physiotherapists and hopefully decrease the referral rate to the orthopaedic surgeons. In this study, only 22.7% of patients were considered as having chronic complaints (i.e. complaints for more than 3 months).^34^ This seems contradictory with the general consensus where PFP is viewed as a chronic complaint persisting for multiple years.^7,9,24,25^ Furthermore, the chronic and nonchronic groups in this study did not differ from each other regarding demographics (i.e. age and sex) and most importantly management

This could possibly be due to a similar duration of complaints before the first presentation in both the chronic and nonchronic PFP groups. However, as mentioned before this information was not available to us. Previous research has found that duration of complaint, a low baseline Kujala Patellofemoral Score^39^ and pain at multiple MSK sites are predictors of a poorer prognosis in patients with PFP.^40^ However, in this current study these predictors were not available. Drawing more attention to these predictors could provide a simple measure for the GP to increase identification of (possible) chronic patients and lead to a more hands on approach in management, for example by proactively planning a check-up consultation or involving physiotherapists early. More research is needed into the efficacy of these measures in chronic patients.

## Conclusion

PFP is a prevalent^9,12^ and impactful^7,24,25^ complaint in children and adolescents with an incidence of 3.4 per 1,000 person years. Peak incidence was at age 13 years for both girls and boys. In general, GP’s adhere to the current Dutch guideline in the management of PFP, with advice being the most administered management strategy followed by referral to the physiotherapist and analgesics. However, more attention should be paid to increase the rate of referral for physiotherapy for exercise therapy because of the aforementioned benefit of supervised muscle strengthening exercises.

At this moment, approximately 30% of the patients have multiple consultations for their complaint. Nevertheless, no difference in age, sex, or treatment was found between chronic and nonchronic patients. It is important to gain more insight into this population because of the long-lasting nature of PFP.^24,25^ Future studies should look into the identifying characteristics and effective treatment of, especially chronic, PFP patients. Furthermore, since this study shows a peak incidence for PFP at age 13 years, subsequent research should already include children and adolescents from that age onwards. Long-term observational studies that include children and adolescents could possibly provide simple measures to earlier identify children at risk to develop chronic complaints.

## Supplementary Material

cmad087_suppl_Supplementary_Table_S1

cmad087_suppl_Supplementary_Checklist

## Data Availability

Data not publically available.
