# Supplementary material for: Patellofemoral pain in general practice: the incidence and management
Source: Fam Pract. 2023 Sep 5;40(4):589–95. doi: 10.1093/fampra/cmad087 (PMC10667070; doi:10.1093/fampra/cmad087)
Supplement: cmad087_suppl_Supplementary_Table_S1 [file cmad087_suppl_supplementary_table_s1.pdf]

Supplementary Table 1: Overview of management policies during all consultations

Policy during consultation	First (N=1030)*	Second (N=306)*	Third (N=116)*	Fourth (N=38)*	Fifth-Ninth (N=25)*	Total (N=1516)*
Advice/Rest/Wait and See	778 (75.5%)	143 (46.7%)	30 (25.9%)	10 (26.3%)	9 (36.0%)	970 (64.0%)
Analgesics	107 (10.4%)	27 (8.8%)	18 (15.5%)	3 (7.9%)	3 (12.0%)	158 (10.4%)
**Referral**						
Imaging	29 (2.8%)	43 (14.1%)	16 (13.8%)	3 (7.9%)	3 (12.0%)	94 (6.2%)
Physiotherapy	274 (26.6%)	106 (34.6%)	34 (29.3)	11 (28.9%)	2 (8.0%)	427 (28.2%)
Orthopedic surgeon	31 (3.0%)	51 (16.7%)	34 (29.3%)	9 (23.7%)	10 (40.0%)	135 (8.9%)
Sports physician	8 (0.8%)	6 (2.0%)	2 (1.7%)	1 (2.6%)	2 (8.0%)	19 (1.3%)
Other (podiatry, therapeutic movement therapy)	16 (1.6%)	3 (1.0%)	3 (2.6%)	0 (0.0%)	0 (0.0%)	22 (1.5%)
*Patients could receive more than one intervention per consultation, therefore the total of patients in these cells does not add up to 100%						

Supplementary Table 1A: Overview of different management policies during all consultations (range 1-9) in the whole population (N=1030)

Policy during consultation	First (N=234)*	Second (N=234)*	Third (N=102)*	Fourth (N=36)*	Fifth-Ninth (N=25)*	Total (N=631)*
Advice/Rest/Wait and See	170 (72.6%)	116 (49.6%)	28 (27.5%)	10 (27.8%)	9 (36.0%)	333 (52.8%)
Analgesics	28 (12.0%)	17 (7.3%)	13 (12.7%)	2 (5.6%)	3 (12.0%)	63 (10.0%)
**Referral**						
Imaging	6 (2.6%)	31 (13.2%)	14 (13.7%)	3 (8.3%)	3 (12.0%)	57 (9.0%)
Physiotherapy	62 (26.5%)	83 (35.5%)	30 (29.4%)	11 (30.6%)	2 (8.0%)	188 (29.8%)
Orthopedic surgeon	6 (2.6%)	38 (16.2%)	30 (29.4%)	8 (22.2%)	10 (40.0%)	92 (14.6%)
Sports physician	2 (0.9%)	1 (0.4%)	2 (2.0%)	1 (2.8%)	2 (8.0%)	8 (1.3%)
Other (podiatry, therapeutic movement therapy)	3 (1.3%)	2 (0.9%)	3 (2.9%)	0 (0.0%)	0 (0.0%)	8 (1.3%)
*Patients could receive more than one intervention per consultation, therefore the total of patients in these cells does not add up to 100%						

Supplementary Table 1B: Overview of different management policies during all consultations (range 1-9) in the chronic population (N=234)

Policy during consultation	First (N=796)*	Second (N=72)*	Third (N=14)*	Fourth (N=2)*	Total (N=884)*
Advice/Rest/Wait and See	608 (76.4%)	27 (37.5%)	2 (14.3%)	0 (0.0%)	637 (72.1%)
Analgesics	79 (9.9%)	10 (13.9%)	5 (35.7%)	1 (50.0%)	95 (10.7%)
**Referral**					
Imaging	23 (2.9%)	12 (16.7%)	16 (13.8%)	0 (0.0%)	37 (4.2%)
Physiotherapy	212 (26.6%)	23 (31.9%)	4 (28.6%)	0 (0.0%)	239 (27.0%)
Orthopedic surgeon	25 (3.1%)	13 (18.1%)	4 (28.6%)	1 (50.0%)	43 (4.9%)
Sports physician	6 (0.8%)	5 (6.9%)	0 (0.0%)	0 (0.0%)	11 (1.2%)
Other (podiatry, therapeutic movement therapy)	11 (1.4%)	1 (1.4%)	0 (0.0%)	0 (0.0%)	12 (1.4%)
*Patients could receive more than one intervention per consultation, therefore the total of patients in these cells does not add up to 100%					

Supplementary Table 1C: Overview of different management policies during all consultations (range 1-4) in the non-chronic population (N=796)
